# Dual tachyarrhythmia: Ventricular fibrillation induced by atrial tachyarrhythmia in a patient with hypertrophic cardiomyopathy

**DOI:** 10.1002/joa3.12980

**Published:** 2023-12-22

**Authors:** Takahiko Kinjo, Shingo Sasaki, Daisuke Horiuchi, Yuji Ishida, Hirofumi Tomita

**Affiliations:** ^1^ Department of Cardiology and Nephrology Hirosaki University Graduate School of Medicine Hirosaki Japan; ^2^ Department of Advanced Management of Cardiac Arrhythmias Hirosaki University Graduate School of Medicine Hirosaki Japan; ^3^ Department of Cardiac Remote Management System Hirosaki University Graduate School of Medicine Hirosaki Japan; ^4^ Department of the Advanced Therapeutics for Cardiovascular Diseases Hirosaki University Graduate School of Medicine Hirosaki Japan

## Abstract

A patient with hypertrophic cardiomyopathy experienced cardiopulmonary arrest. An automated external defibrillator administered defibrillation for ventricular fibrillation (A). The pacemaker recorded atrial tachycardia with a rapid ventricular response before the patient collapsed (B). After a few minutes, the pacemaker records dual tachyarrhythmia, characterized by the simultaneous presence of ventricular fibrillation (VF) and atrial fibrillation (AF) (C). This case demonstrates that VF induced by atrial tachyarrhythmia could contribute to AF‐related sudden cardiac death.

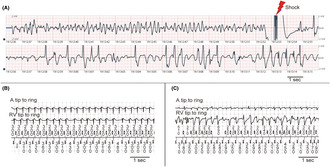

Atrial tachyarrhythmias, including atrial fibrillation (AF) and atrial tachycardia (AT), are strongly associated with hypertrophic cardiomyopathy (HCM).^1^ In patients with HCM, AF at the time of implantable cardioverter‐defibrillator (ICD) implantation is a risk factor for appropriate therapy.^2^ Furthermore, AF has been thought to be related to sudden cardiac death (SCD)^3^; however, its mechanism remains unclear. This article describes a case of a patient with HCM who presented ventricular fibrillation (VF) induced by atrial tachyarrhythmia.

An 81‐year‐old woman with nonobstructive HCM and a history of four times previous ablations for paroxysmal AF and AT was admitted to our hospital because of sick sinus syndrome. A dual‐chamber pacemaker was implanted since the patient had no major high‐risk findings for SCD.^1^ The absence of these factors included a maximum wall thickness of 19 mm, no nonsustained ventricular tachycardia, no history of syncope, and no family history of SCD. She had been treated with a β‐blocker (bisoprolol 2.5 mg/day); It was discontinued preoperatively but resumed after implantation. Six days after implantation, she was scheduled to be discharged owing to a good course; therefore, the monitoring electrocardiogram was removed. However, the patient experienced cardiopulmonary arrest (CPA) in the hospital, which required cardiopulmonary resuscitation (CPR). An automated external defibrillator detected VF; accordingly, the patient underwent defibrillation (Figure 1A). AT occurred before the syncope, suggesting that AT with rapid ventricular response led to syncope (Figures 1B and 2A). Thereafter, irregular tachycardia of the atrial and ventricle was recorded, which coincided with the CPR time (Figures 1C,D and 2B). We defined this waveform as dual tachyarrhythmia, characterized by the simultaneous presence of VF and AF. Dual tachyarrhythmia was determined because the waveforms were recorded while the patient was in CPA, the electrograms recorded on the atrial and ventricular leads differed for each successive beat, and the subsequent record of VF on the surface electrogram. We concluded that AT induced VF by scatter plot (Figure 2B), although the exact onset timing of VF may be impaired because of the possibility of under‐sensing in the ventricular leads. After successful treatment, the device was upgraded to the transvenous ICD; moreover, the patient started amiodarone (200 mg/day for a year and reduced to 100 mg/day) for rhythm control. After 2 years, the patient has not yet experienced a recurrence of AT/AF and VF.

**FIGURE 1:**
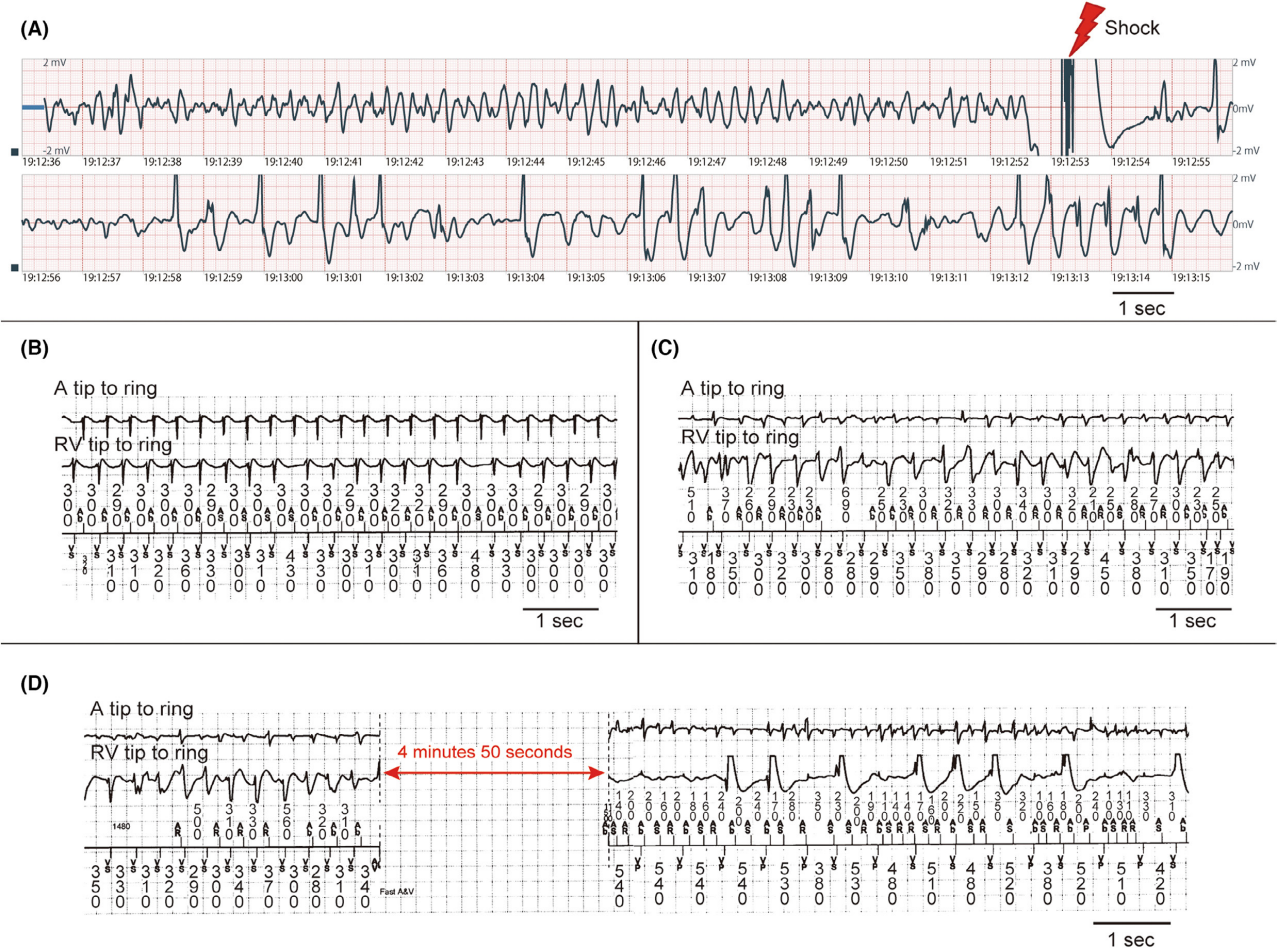
Electrocardiogram records of the automated external defibrillator (A) and pacemaker (B–D). (A) Defibrillation treatment was administered to ventricular fibrillation using an automated external defibrillator. (B) An electrogram record before the patient initiated cardiopulmonary resuscitation (CPR). Atrial tachycardia with a rapid ventricular response was recorded. After the onset of atrial tachycardia, the physician examined the patient and determined that she was in cardiopulmonary arrest. (C) An electrogram record during CPR is being performed. Dual tachyarrhythmia, which was characterized by irregular atrial and ventricular tachycardia, was observed. (D) An electrogram record following Figure 1C. No electrogram was recorded during the time of shock. The atrial fibrillation persisted after shock. CPR, cardiopulmonary resuscitation.

**FIGURE 2:**
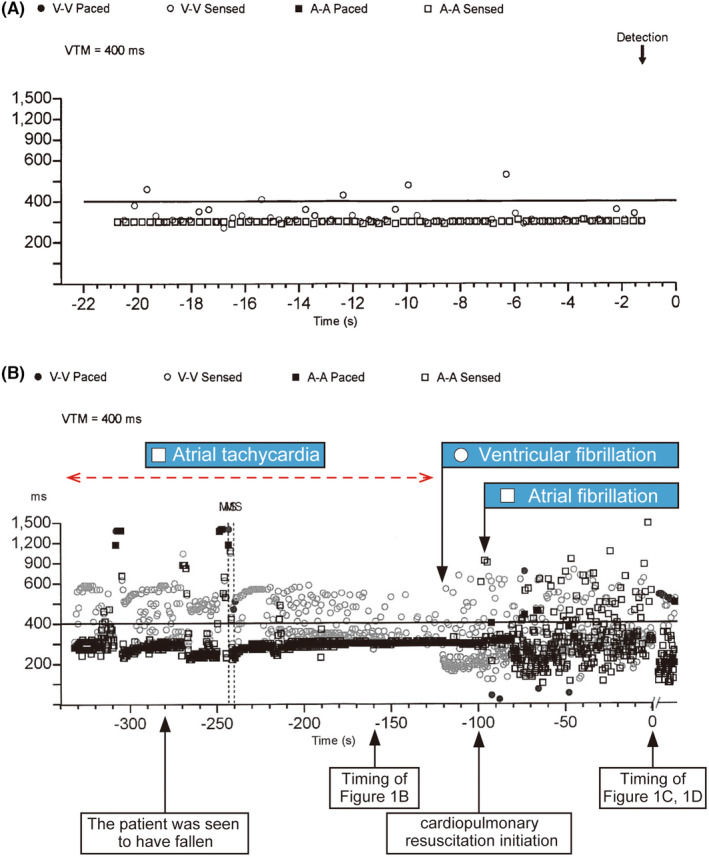
Scatter plot of coupling intervals before detecting atrial tachycardia (A) or dual tachyarrhythmia (B). (A) The atrium is already in a state of regular tachycardia (white dots) before the detection, accompanied by a rapid ventricular response (white square). (B) The coupling interval shortening and variability of the ventricle, which indicates ventricular fibrillation, was observed 120 s before the detection. The coupling interval shortening and variability of the atrium, which indicates atrial fibrillation, was observed 100 s before detection. Atrial tachycardia induced ventricular fibrillation and subsequently changed from atrial tachycardia to fibrillation.

A clinical implication of this report is that atrial tachyarrhythmia with a rapid ventricular response can induce VF in patients with HCM, which could involve an oxygen demand/supply mismatch because of tachycardia. This suggests that VF is one of the mechanisms underlying AF‐related SCD. As shown in Figure 3, the short PQ interval during sinus rhythm and short coupling interval during AF suggested the good conduction property of the atrioventricular (AV) node. We would better be aware that AT/AF can induce VF in such patients, and aggressive prophylactic antiarrhythmic medical therapy or AV node ablation may be considered. The treatment strategy of dual tachyarrhythmia remains to be elucidated; however, the patient would require AV node ablation if AT/AF was uncontrollable.

**FIGURE 3:**
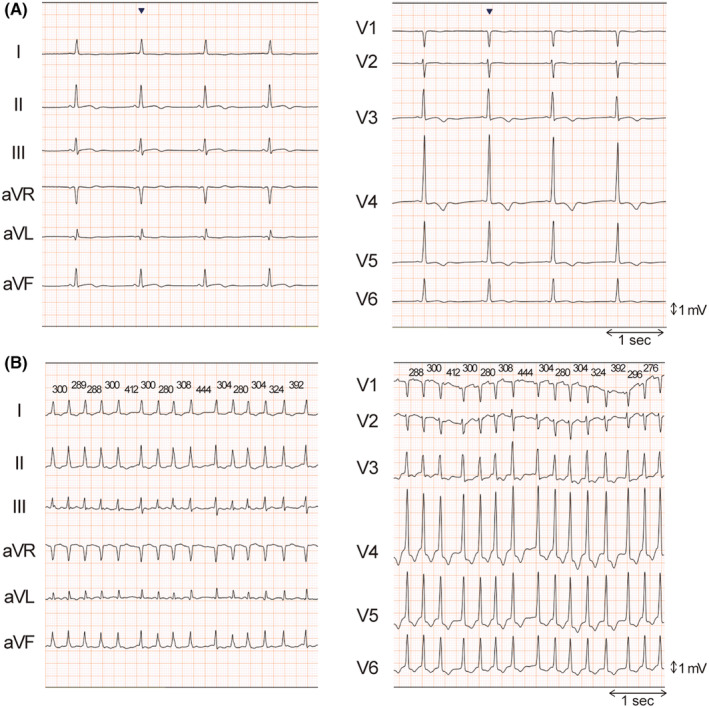
Twelve‐lead electrocardiograms during sinus rhythm (A) and atrial fibrillation (B) before this event. (A) PQ interval is 112 ms. (B) The shortest coupling interval during atrial fibrillation is 288 ms.

Another clinical implication is that dual tachyarrhythmia may be underrecognized. VF immediately results in SCD; confirming its presence in patients without cardiac implantable electronic devices is difficult. In patients undergoing transvenous ICD implantation, ventricular tachycardia induced by atrial tachyarrhythmia has been reported,^4^ making it challenging to distinguish between appropriate and inappropriate therapy. There have been studies on inappropriate shock for AF^5^; however, dual tachyarrhythmia has yet to be described.

In conclusion, this case demonstrates that VF induced by atrial tachyarrhythmia could contribute to AF‐related SCD in patients at risk of circulatory collapse or myocardial ischemia because of tachycardia, such as HCM.

## FUNDING INFORMATION

This report received no specific grant from public, commercial, or not‐for‐profit funding agencies.

## CONFLICT OF INTEREST STATEMENT

Dr. Shingo Sasaki received a research grant from Boston Scientific Japan Co., Ltd. and is a concurrent associate professor of the Department of Advanced Management of Cardiac Arrhythmias and the Department of Cardiac Remote Management System. Dr. Yuji Ishida is an assistant professor of the Department of Cardiac Remote Management Systems, which is an endowment department supported by BIOTRONIK Japan Co., Ltd. Dr. Hirofumi Tomita is a concurrent professor of the Department of Advanced Management of Cardiac Arrhythmias, the department of Cardiac Remote Management System, and the department of the Advanced Therapeutics for Cardiovascular Diseases, which is an endowment department supported by Boston Scientific Japan Co., Ltd. Dr. Tomita also received a research grant from Abbott Medical Japan LLC. Other authors have no relevant disclosures.

## ETHICS STATEMENT

Informed consent was obtained from the patient to publish this case report.

## Data Availability

Data sharing does not apply to this article because no datasets were generated or analyzed during the current report.
